# Investigation of Surface Morphology and Roughness of Dental Implant Platform and Abutment

**DOI:** 10.3390/dj14090585

**Published:** 2026-09-10

**Authors:** Teodora Filipova, Stefan Peev, Stanislav Mitkov

**Affiliations:** 1 Department of Periodontology and Dental Implantology, Faculty of Dental Medicine, Medical University of Varna, 9002 Varna, Bulgaria; stefan.peev@mu-varna.bg; 2 Department of Dental Materials Science and Prosthetic Denal Medicine, Medical University of Varna, 9002 Varna, Bulgaria; stanislav.mitkov@mu-varna.bg

**Keywords:** dental implant platform, dental abutment, surface roughness, scanning electron microscopy (SEM)

## Abstract

**Purpose**: This study investigated the surface morphology and roughness of the dental implant platform and abutment at their junction interface, comparing parameters before and after mechanical assembly (screwing). **Methods**: The experimental specimens comprised 40 implants from the TBR, Bone Level M System (TBR, France) with a diameter of 4.7 mm and a length of 10 mm, and 40 matching abutments from the same system (Titanium Standard Abutment, TBR, France) with a diameter of 4 mm and a gingival height of 3 mm. All components were fabricated from Grade 4 titanium. The implants and abutments were divided into two equal groups. In the first group (control), 20 implants and 20 abutments remained unassembled. In the second group, 20 implants were coupled with 20 abutments and tightened to the manufacturer-recommended torque. To achieve this, the implants were stabilized with pliers to prevent rotation, and the abutments were screwed using a screwdriver and a torque wrench at 30 N.cm. Subsequently, the abutments were unscrewed counterclockwise. Surface evaluations were conducted using scanning electron microscopy (SEM) and 3D surface modeling. **Results**: The obtained results demonstrate a statistically significant reduction in the parameters reflecting the roughness of the implant platform. Based on the analysis, a pronounced decrease in the average roughness (Ra, Rq) of the abutment was observed (*p* < 0.001). Furthermore, it was established that the implant platform exhibits nearly twice the surface roughness of the abutment even in its baseline state. **Conclusions**: Within the limitations of this in vitro study, a single tightening and removal procedure was associated with measurable changes in the roughness parameters and surface morphology of the implant platform and abutment. These findings indicate microtopographic adaptation at the conical interface under the tested conditions; however, biomechanical reliability and resistance to bacterial micro-leakage were not evaluated.

## 1. Introduction

The long-term success of dental implants depends not only on their osseointegration with the surrounding bone but also on the integrity and stability of the implant–abutment interface. Among the key microstructural features influencing both biological and mechanical outcomes is surface roughness—a parameter that governs cell adhesion, microbial colonization, and mechanical interlocking at the interface between implant components and surrounding tissues [1].

The surface roughness of a material quantifies topographic deviations from an ideal geometric form and is typically assessed from variation in the surface normal vector [2]. Material properties and the specific manufacturing technique determine the overall degree of roughness. Accordingly, surface roughness results directly from fabrication processes such as casting, grinding, or cutting [3].

Surface roughness of the implant platform and abutment may influence osseointegration, soft tissue integration, bacterial colonization, peri-implant bone stability, and the long-term performance of dental implants.

According to the American Academy of Implant Dentistry, osseointegration is defined as the “contact established without interposition of nonbone tissue between normal remodeled bone and an implant entailing a sustained transfer and distribution of load from the implant to and within the bone tissue” [4]. Clinically, osseointegration manifests as rigid stability and functional ankylosis of the implant within bone tissue [5]. Early osseointegration is influenced by the interplay among mechanical factors, chemical composition, and the specific microroughness and nanoroughness of the implant surface [6].

Clinical and in vitro investigations are therefore needed to clarify the relationship between dental-implant surface roughness and osseointegration. Tissue responses to biomaterials are influenced by the nature and texture of the implant surface. Compared with smooth substrates, textured implant surfaces provide a larger surface area for bone apposition and may facilitate tissue ingrowth [7,8].

Surface roughness, often quantified by the arithmetical mean roughness (Ra), has a dual role in implantology. Moderately rough surfaces (Ra between 1.0 and 2.0 µm) on the implant body have been reported to enhance osteoblast adhesion and promote osseointegration by increasing surface energy and wettability [9,10]. By contrast, roughness on the implant platform and abutment, which may be exposed to the oral environment, may increase susceptibility to bacterial colonization, biofilm formation, and peri-implant inflammation [11,12].

In a systematic review, Wennerberg and Albrektsson (2009) [10] classified implant surfaces based on their topography:

- Smooth: Ra < 0.5 µm (associated with low cell adhesion and limited bone contact).
- Minimally rough: Ra = 0.5 µm (improved response compared to smooth surfaces, yet remaining sub-optimal).
- Moderately rough: Ra = 1.0–2.0 µm (optimal for osteoblast activity and bone ingrowth).
- Rough: Ra > 2.0 µm (potentially impairing cell spreading and elevating the risk of peri-implant inflammation).

Furthermore, the authors concluded that moderately rough surfaces (Ra 1.5 µm) may optimize osseointegration by enhancing osteoblast attachment and proliferation, thereby providing favorable conditions for neo-osteogenesis [10].

Buser et al. (2002) [13] reported that rougher surfaces were associated with greater bone-to-implant contact (BIC); sandblasted and acid-etched (SLA) surfaces increased early BIC and reduced healing times. Conversely, highly rough surfaces may promote bacterial colonization and inflammatory responses, particularly in the crestal area. These observations underscore the need to balance surface characteristics that support bone healing against those that may favor microbial plaque retention.

The abutment traverses the oral mucosa and establishes a transmucosal connection between the external oral cavity and underlying bone. The peri-implant soft-tissue barrier surrounding the abutment is important for the long-term success of oral implants. Clinical studies have reported a positive association between implant–abutment surface roughness (Ra) and supragingival and subgingival plaque attachment [14,15].

According to Wennerberg and Albrektsson (2009) [10], and the ISO 25178 standards, dental abutment surfaces are classified as follows:

- Smooth: Ra < 0.5 µm (minimal plaque accumulation; ideal for transmucosal abutments).
- Minimally rough: Ra = 0.5–1.0 µm (some cell adhesion benefits, but accompanied by an increased biofilm risk).
- Moderately rough: Ra = 1.0–2.0 µm (not recommended for abutments; more suitable for implant threads).
- Rough: Ra > 2.0 µm (unsuitable for abutments due to a high plaque retention risk).

Smooth abutment surfaces (Ra < 0.5 µm) have been associated with reduced plaque retention and bacterial colonization, peri-implant soft-tissue stability, and epithelial cell migration and adhesion. A potential disadvantage is reduced fibroblast attachment strength. By contrast, rougher abutment surfaces (Ra > 0.5 µm) may enhance fibroblast proliferation but have also been associated with greater biofilm accumulation and a higher risk of peri-implant mucositis and peri-implantitis [16,17,18].

Although substantial research has addressed optimization of the intraosseous implant surface, fewer studies have examined the topography of the implant platform and transmucosal abutment. These regions form the coronal portion of the implant system and contribute to soft-tissue integration, sealing, and defense against microbial penetration at the implant–abutment junction (IAJ) [18,19,20].

Mechanical connection of the abutment to the implant may alter surface roughness through micromotion, fretting wear, or titanium-particle release at the interface [21]. Such changes may compromise connection stability and contribute to peri-implant bone loss or soft-tissue inflammation, particularly if roughness exceeds thresholds associated with bacterial adhesion [22].

Profilometric analyses using stylus profilometry, white-light interferometry, or scanning electron microscopy (SEM) have shown that surface roughness may be altered by torque application, repeated screwing and unscrewing, or clinical procedures such as implantoplasty [23]. These alterations may affect both the mechanical behavior of the implant–abutment interface and the biological response of the peri-implant tissues.

Accordingly, the aim of this in vitro study was to compare the surface morphology and roughness of the implant platform and abutment before and after a single mechanical assembly, tightening, and removal procedure.

## 2. Materials and Methods

The present study investigated implants from the TBR Bone Level M system (TBR, Toulouse, France; Cat. No. 12/1144, REF-M510). The implants had a diameter of 4.7 mm and a length of 10 mm and were manufactured from commercially pure Grade 4 titanium in accordance with ISO 5832-2. The corresponding prosthetic components were Titanium Standard Abutments (TBR, France; Cat. No. 12/0013, REF-M-MT530), with a diameter of 4 mm and a gingival height of 3 mm, also fabricated from Grade 4 titanium. The implant–abutment interface consisted of a 4° conical connection combined with an octagonal indexing feature.

The implants and abutments were allocated into two experimental groups:

- Group 1 (control group): Twenty implants and twenty abutments were used without being connected or tightened together.
- Group 2: Twenty implants and twenty abutments were assembled and tightened according to the manufacturer’s recommended torque protocol. To prevent rotational movement during tightening, the implants were stabilized using forceps, while the abutments were secured using a screwdriver and a calibrated torque wrench to a torque of 30 N·cm. Subsequently, the abutments were removed by unscrewing them counterclockwise using the same instrumentation.

For the purpose of this study, “implant platform” refers to the coronal seating surface of the implant at the implant–abutment interface. Surface roughness analysis was performed exclusively in this connection area. During torque application, the implant was stabilized by gripping the external implant body only, without contact with the analyzed platform surface.

Prior to the evaluation of implant platform surface roughness, specimen preparation was performed according to a specifically developed protocol.

A dedicated preparation method and custom-made device were developed to produce hard-section specimens suitable for microscopic evaluation of internal implant-surface roughness. The steel fixture comprised three components: a cylindrical housing, an upper section, and a lower section. The working surface of the upper component had a convex cone, whereas the lower component contained a corresponding concave cone, enabling precise axial positioning of the implant. A 20 mm-diameter PVC tube defined the working chamber and contained an access opening through which the specimens were embedded in resin using a pipette.

The specimen preparation protocol consisted of the following steps:

1. Fixation of the implant within the preparation device.
2. Embedding in epoxy resin.
3. Resin polymerization and specimen retrieval from the device.
4. Sectioning of the specimen along the longitudinal axis of the implant.
5. Rinsing with water, drying, and ultrasonic cleaning in isopropanol for 15 s.

**Figure 1:**
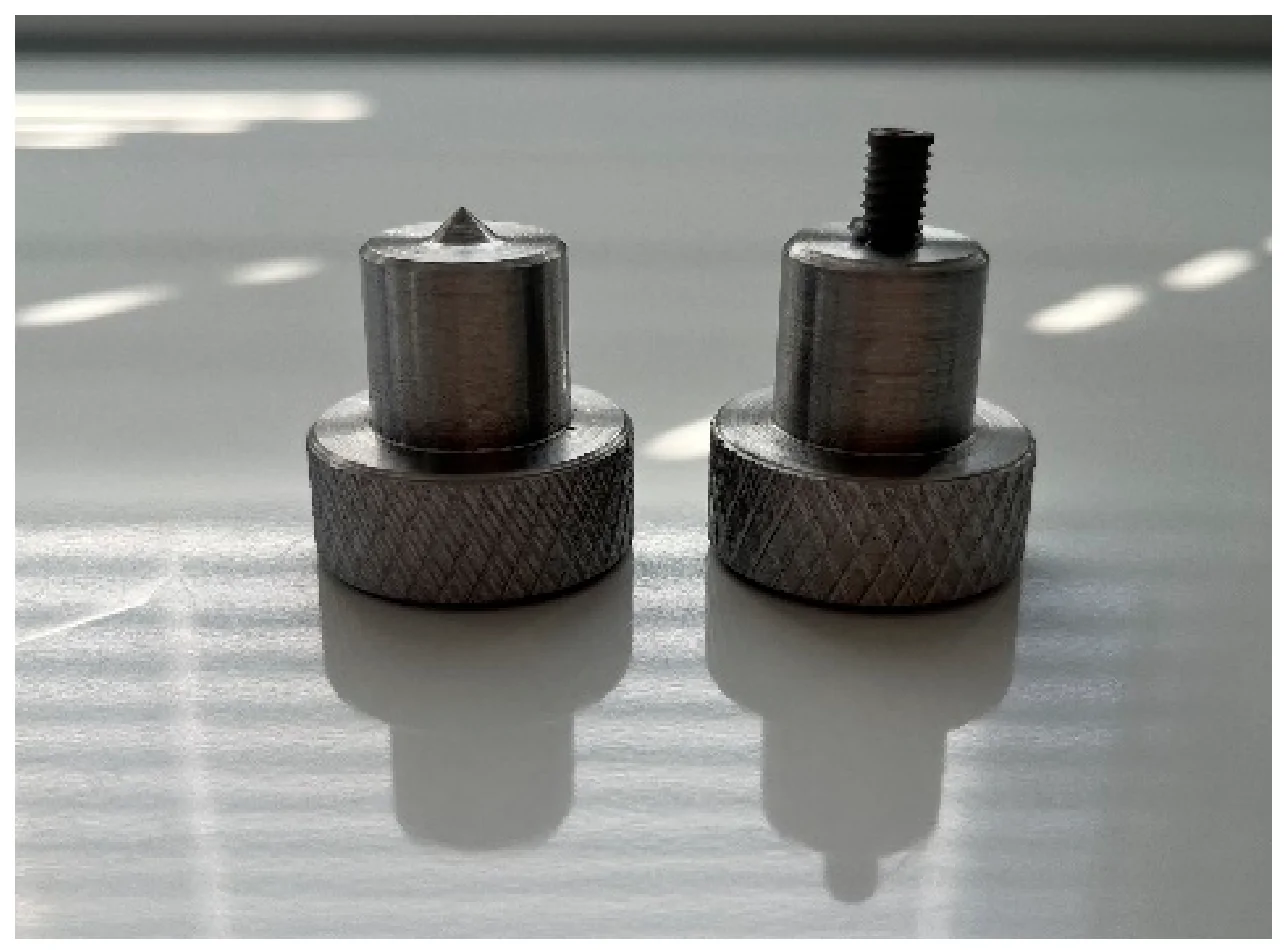
The implant was positioned in the lower section of the specimen preparation device.

**Figure 2:**
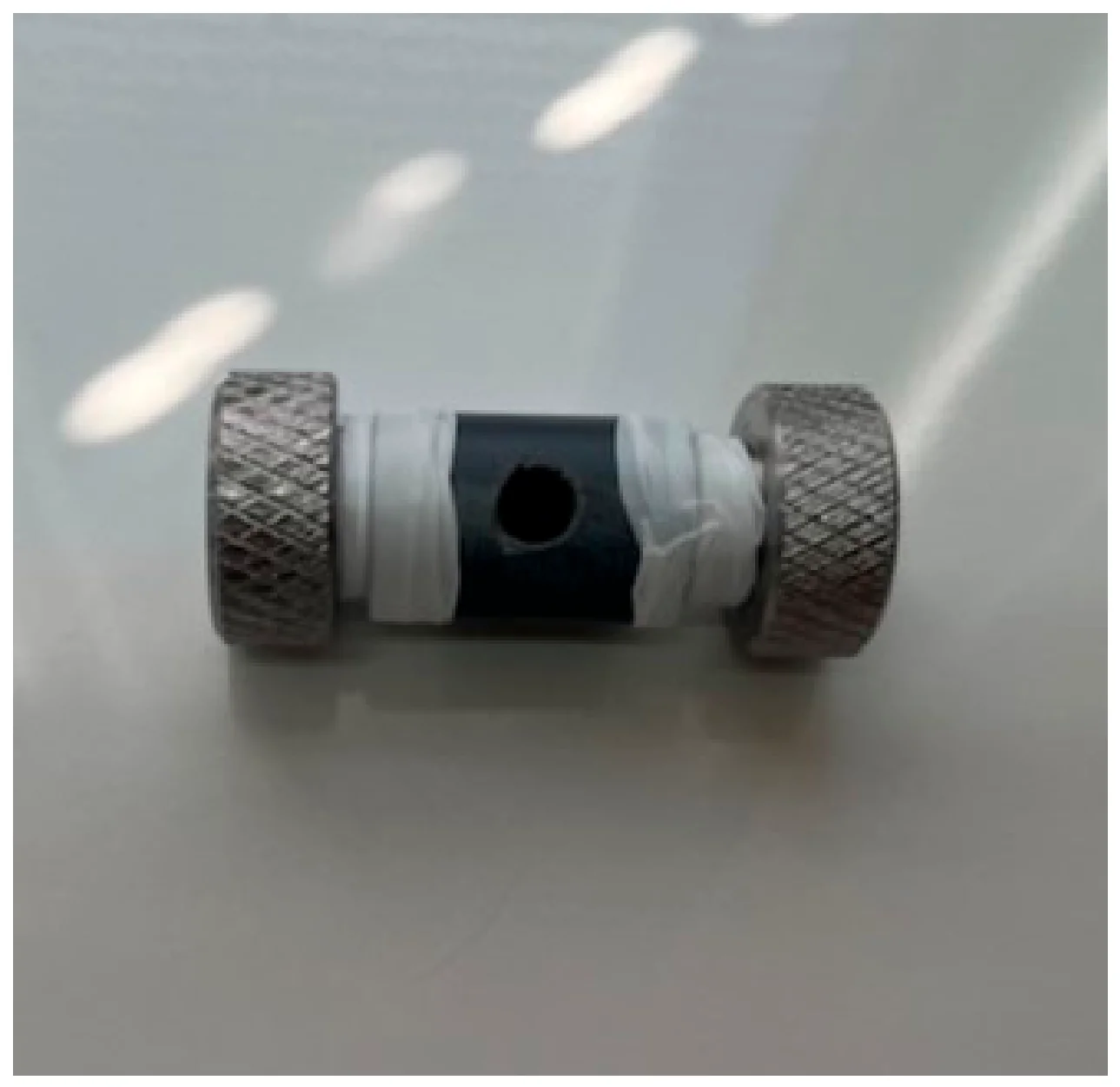
PTFE sealing tape wrapped around both ends of the PVC tube to prevent resin leakage during polymerization.

The implant was positioned within the lower conical seat of the device (Figure 1). Subsequently, the PVC tube was installed, followed by placement of the upper centering component, which ensured accurate alignment of the specimen within the geometric center of the system. To prevent uncontrolled resin leakage before polymerization, all contact areas between the metallic components of the fixture and the PVC tube were hermetically sealed using polytetrafluoroethylene (PTFE) tape (Figure 2).

Epoxy Resin Embedding

**Figure 3:**
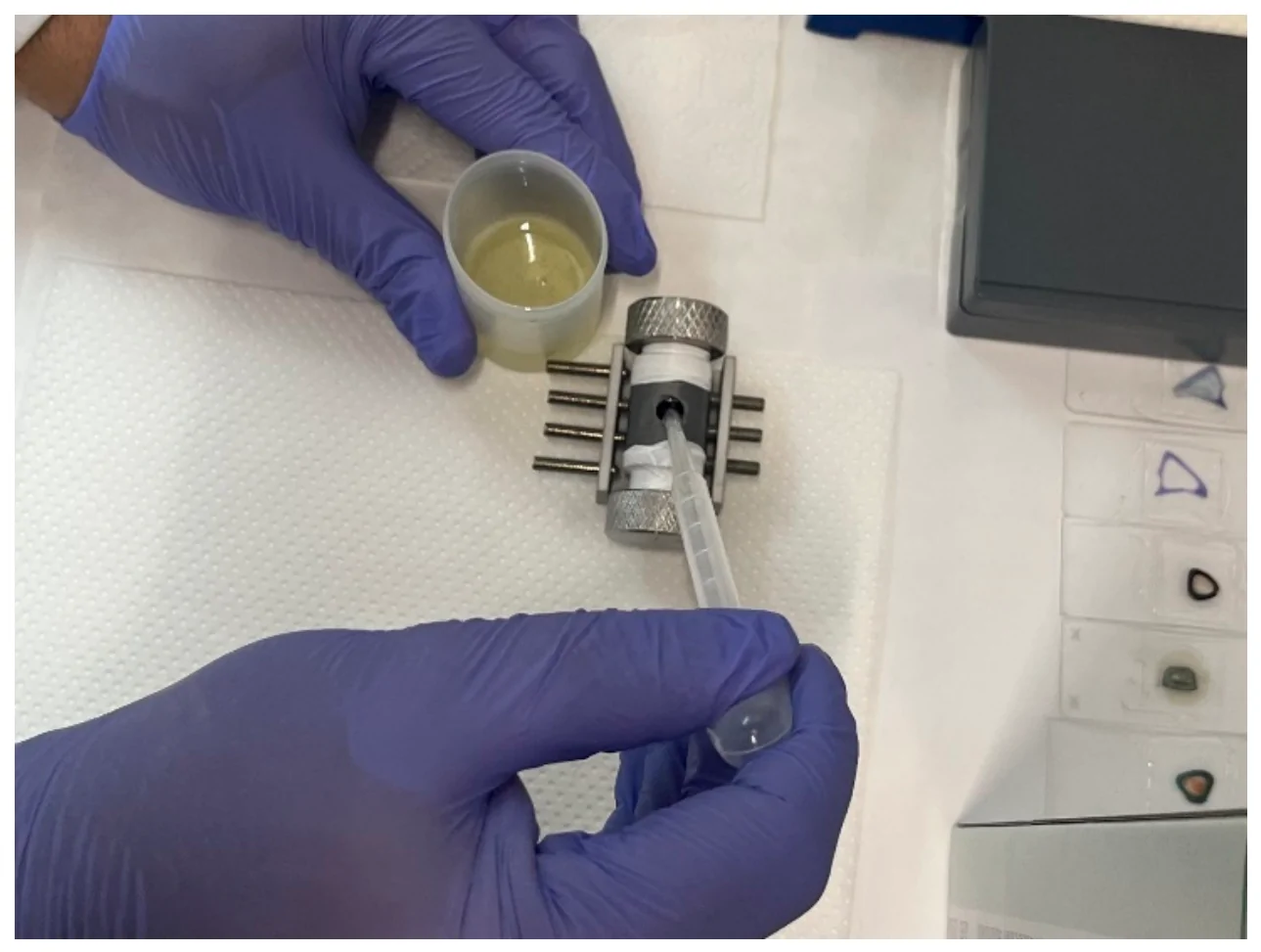
Embedding of the implant in epoxy resin.

Epoxy resin (Albedo Nova) was introduced through the opening in the PVC tube (Figure 3). The resin was prepared according to the manufacturer’s specifications.

The resin was allowed to polymerize completely for 5 days.

**Figure 4:**
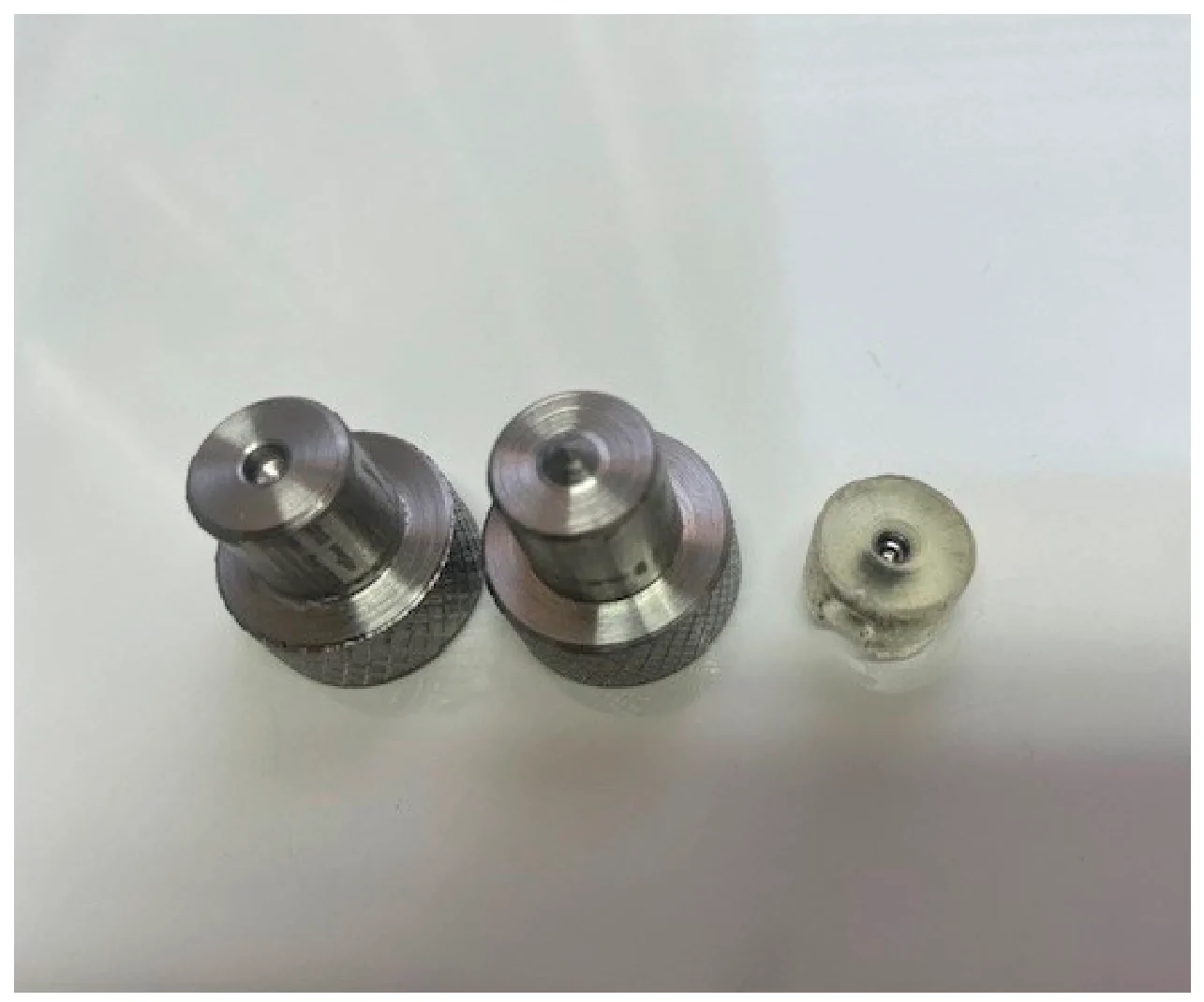
Implant specimen after complete resin polymerization and removal from the preparation device.

After complete curing, the upper and lower components of the fixture, together with the PVC tube, were removed, and the embedded specimen was released (Figure 4).

Sectioning Procedure

**Figure 5:**
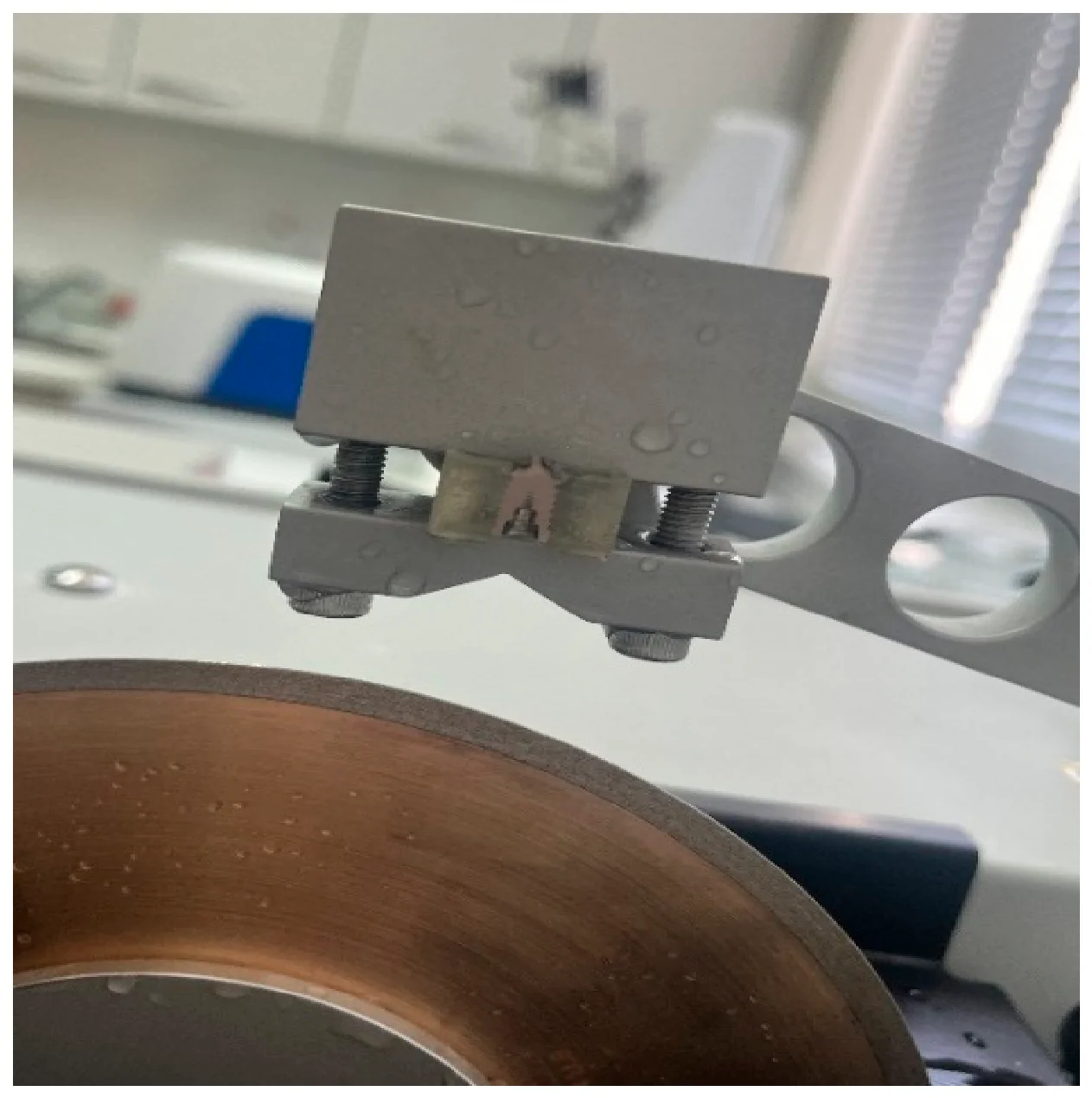
Longitudinally sectioned implant specimen.

The polymerized blocks were sectioned precisely along the longitudinal (central) axis of the implant using an Isomet 1000 precision metallographic cutting machine (Buehler Ltd., Lake Bluff, IL, USA). Sectioning was performed using a diamond cutting disc operating at low rotational speed under continuous water cooling. This procedure minimized thermal damage to the titanium interface and prevented distortion of the surface profile (Figure 5).

Cleaning Procedure

**Figure 6:**
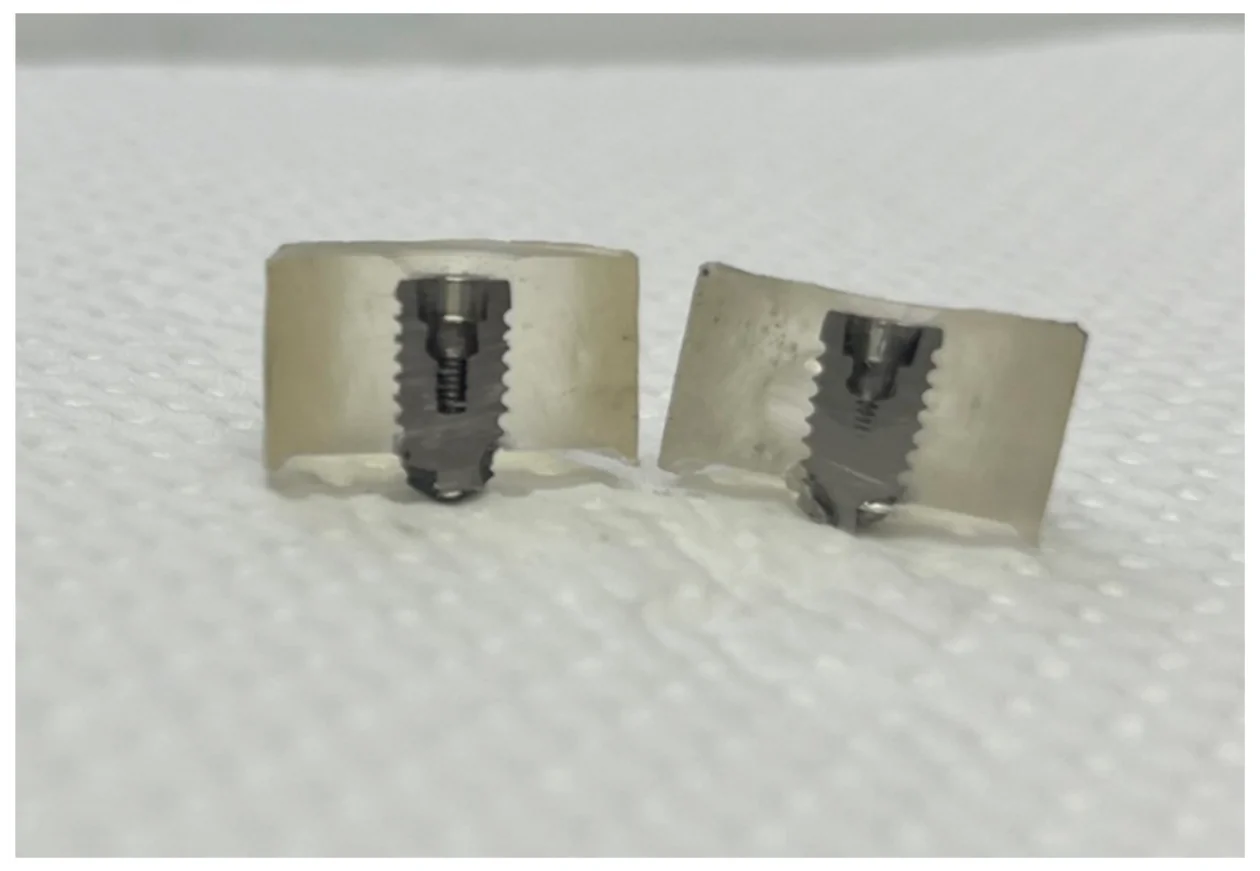
Prepared specimen for investigation.

Following sectioning, the resulting hard-section specimens were thoroughly rinsed with distilled water to remove metallic and polymeric debris and subsequently dried under filtered airflow. To achieve complete degreasing and removal of microscopic surface contaminants, the specimens were subjected to ultrasonic cleaning in pure isopropanol using a Branson 3800 ultrasonic bath (Branson Ultrasonics, Danbury, CT, USA) for 15 s, followed by final drying in a dust-free environment (Figure 6).

The abutments used in the study did not require any additional specimen preparation procedures.

Surface Roughness Analysis

Quantitative and qualitative characterization of the prepared titanium interface surfaces was performed using a Gemini 2 SEM 460 scanning electron microscope (Carl Zeiss AG, Oberkochen, Germany).

To generate high-resolution topographical maps without destructive sample preparation, the microscope operated in variable-pressure backscattered electron mode, employing a specialized VP BSD (Variable Pressure Backscattered Detector, Carl Zeiss AG, Oberkochen, Germany). Three-dimensional surface reconstruction and roughness calculations were performed using the proprietary 3D Surface Modelling software Ver. 2.0.33 (Zeiss), which processes signals acquired from the four detector quadrants.

For comprehensive mathematical and physical characterization of the surface microtopography, the following descriptive amplitude and spatial parameters were recorded and analyzed in accordance with ISO 25178 standards for surface texture characterization:

- Ra (Arithmetic Mean Roughness): The arithmetic mean of the absolute deviations of the profile heights from the mean line within the evaluated length.
- Rq (Root Mean Square Roughness): The square root of the mean of the squared profile deviations, particularly sensitive to pronounced peaks and valleys.
- Rp (Maximum Peak Height): The distance from the mean line to the highest peak of the profile within the assessment length.
- Rv (Maximum Valley Depth): The distance from the mean line to the deepest valley of the profile within the evaluated region.
- Rsk (Skewness): A measure of profile asymmetry relative to the mean line. Negative values (Rsk < 0) indicate a valley-dominated surface with greater load-bearing characteristics, whereas positive values (Rsk > 0) indicate a peak-dominated surface morphology.
- Rku (Kurtosis): A descriptor of the sharpness of the height distribution. A value of Rku = 3 corresponds to a normal (Gaussian) distribution. Values Rku > 3 indicate the presence of sharp peaks and deep valleys, whereas Rku < 3 reflects a surface characterized by rounded summits and broader surface features.

## 3. Results

Descriptive statistics were calculated for all roughness parameters. Before-and-after comparisons were performed using paired *t*-tests for parametric data and Wilcoxon signed-rank tests for non-parametric data. Statistical significance was set at *p* < 0.05.

Table 1 presents statistical comparison of the surface roughness parameters of the implant platform before and after tightening to the abutment. Ra decreased from 0.777 µm to 0.650 µm (*p* = 0.007). The largest absolute change was observed for Rv, which decreased by 0.301 µm (*p* = 0.002). Under the study conditions, these changes indicate reduced mean roughness and maximum valley depth of the implant platform after tightening; they are consistent with surface smoothing but do not directly demonstrate plastic deformation or filling of microcavities.

Rp did not change significantly (*p* = 0.18). Thus, the study did not detect a significant change in maximum peak height; Rp alone does not establish penetration of implant micropeaks into the abutment.

Rsk changed from −0.367 to −0.142 (*p* = 0.047), indicating that the initially negative skewness moved toward a more symmetric distribution after tightening. Rku increased from 1.92 to 2.50, consistent with a shift from a relatively flat toward a more peaked profile; however, this change was not statistically significant (*p* = 0.118).

**Figure 7:**
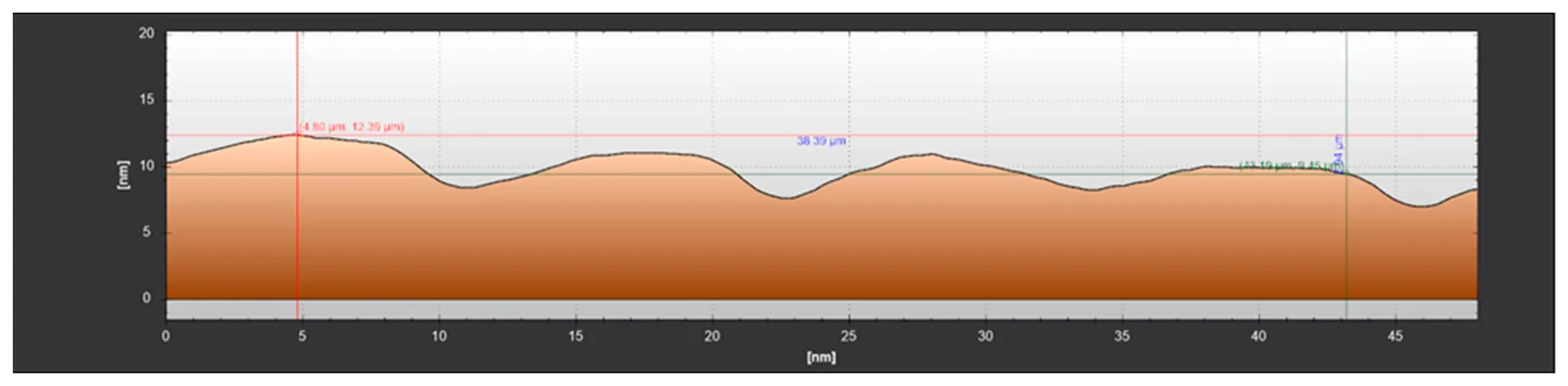
Surface roughness of the implant platform before tightening the abutment.

**Figure 8:**
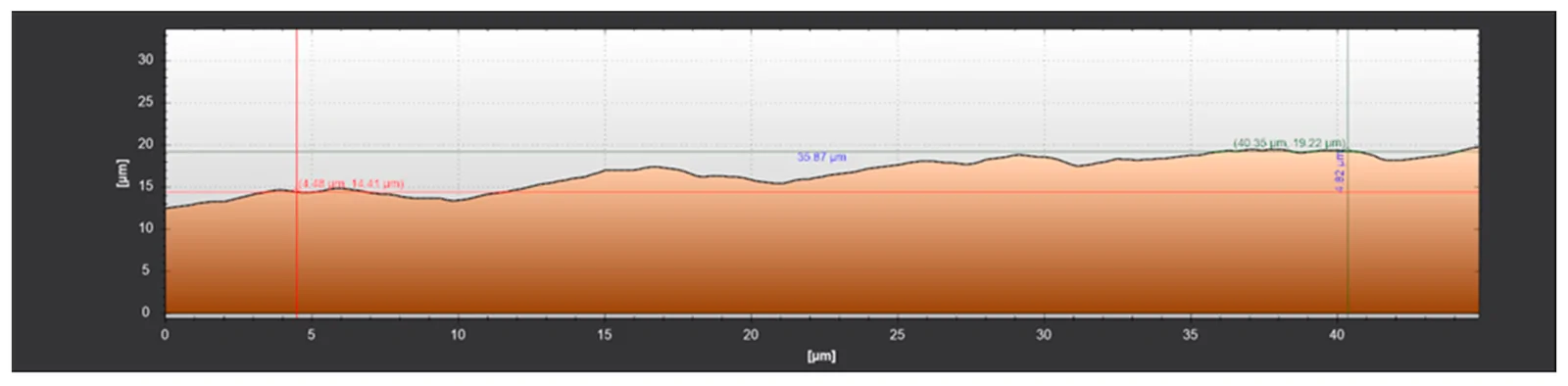
Surface roughness of the implant platform after tightening the abutment.

These patterns are illustrated in Figure 7 and Figure 8 which show implant-platform surface roughness before and after abutment tightening.

**Figure 9:**
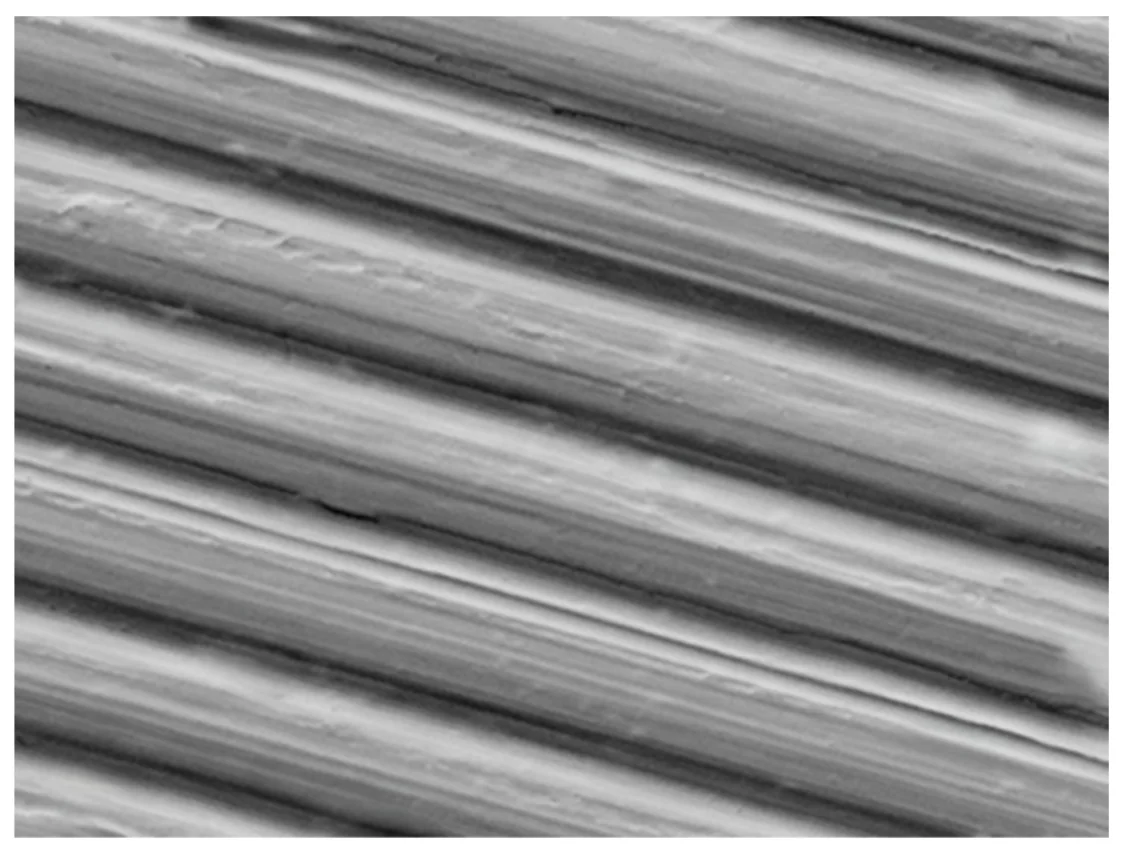
SEM image of the implant platform before tightening the abutment.

**Figure 10:**
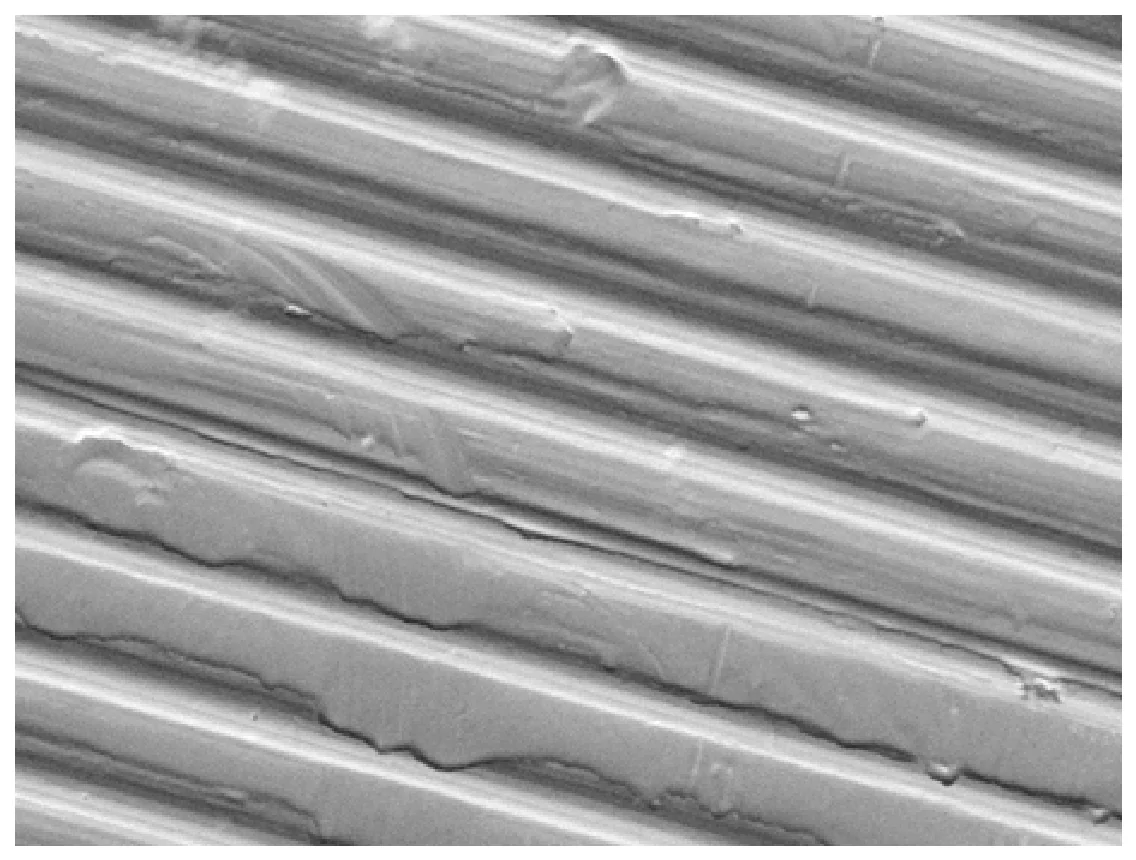
SEM image of the implant platform after tightening the abutment.

SEM images obtained before and after torque application (Figure 9 and Figure 10) qualitatively show reduced micro-roughness and smoothing of the implant-platform surface.

Table 2 presents statistical comparison of the surface roughness parameters of the abutment before and after tightening to the implant. Abutment Ra decreased from 0.437 µm to 0.291 µm (*p* < 0.001). The reported Cohen’s d was 1.85. Compared with the implant platform, the larger decrease in abutment Ra indicates greater smoothing under the tested conditions; differences in material hardness and contact geometry were not evaluated.

For the abutment, Rp decreased by 0.264 µm (*p* < 0.001), whereas Rv increased from 1.167 µm to 1.297 µm (*p* = 0.001). These findings show simultaneous reduction in maximum peak height and increase in maximum valley depth after tightening; they do not, by themselves, establish crushing or deformation of microtips.

The Rku parameter increased from 2.467 µm to 3.790 µm (*p* < 0.001), crossing the value of 3.0 and indicating a shift from a platykurtic to a leptokurtic height distribution. This change describes the shape of the measured profile distribution but does not, by itself, demonstrate intense friction or a specific deformation mechanism.

**Figure 11:**
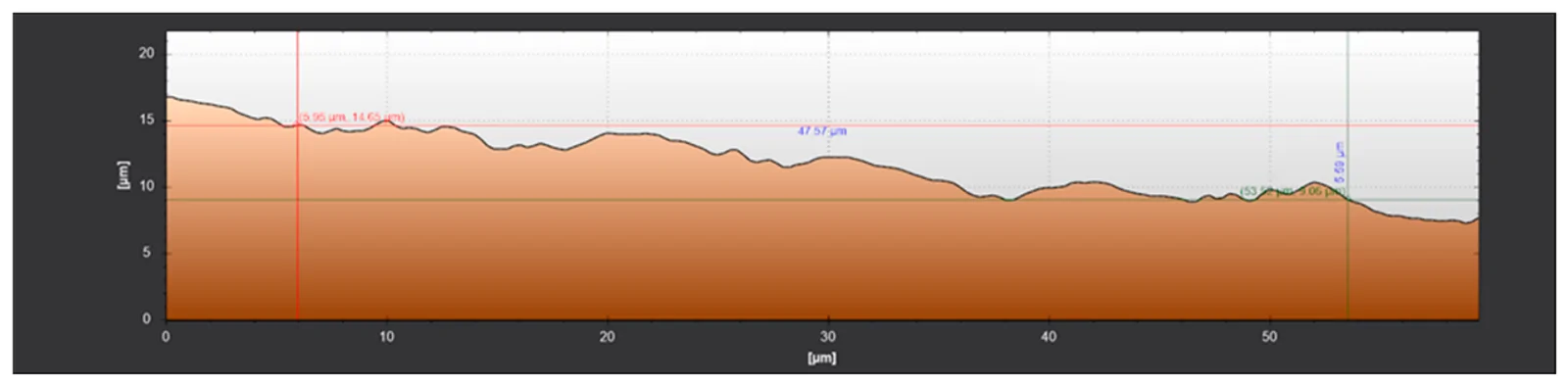
Surface roughness of the abutment before tightening to the implant.

**Figure 12:**
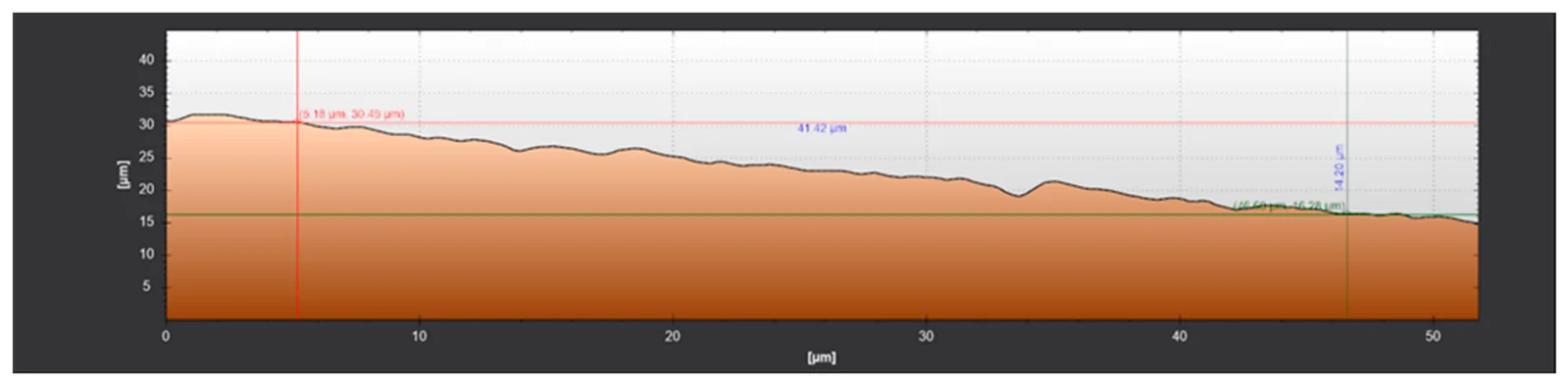
Surface roughness of the abutment after tightening to the implant.

The abutment roughness data before and after tightening are illustrated in Figure 11 and Figure 12.

**Figure 13:**
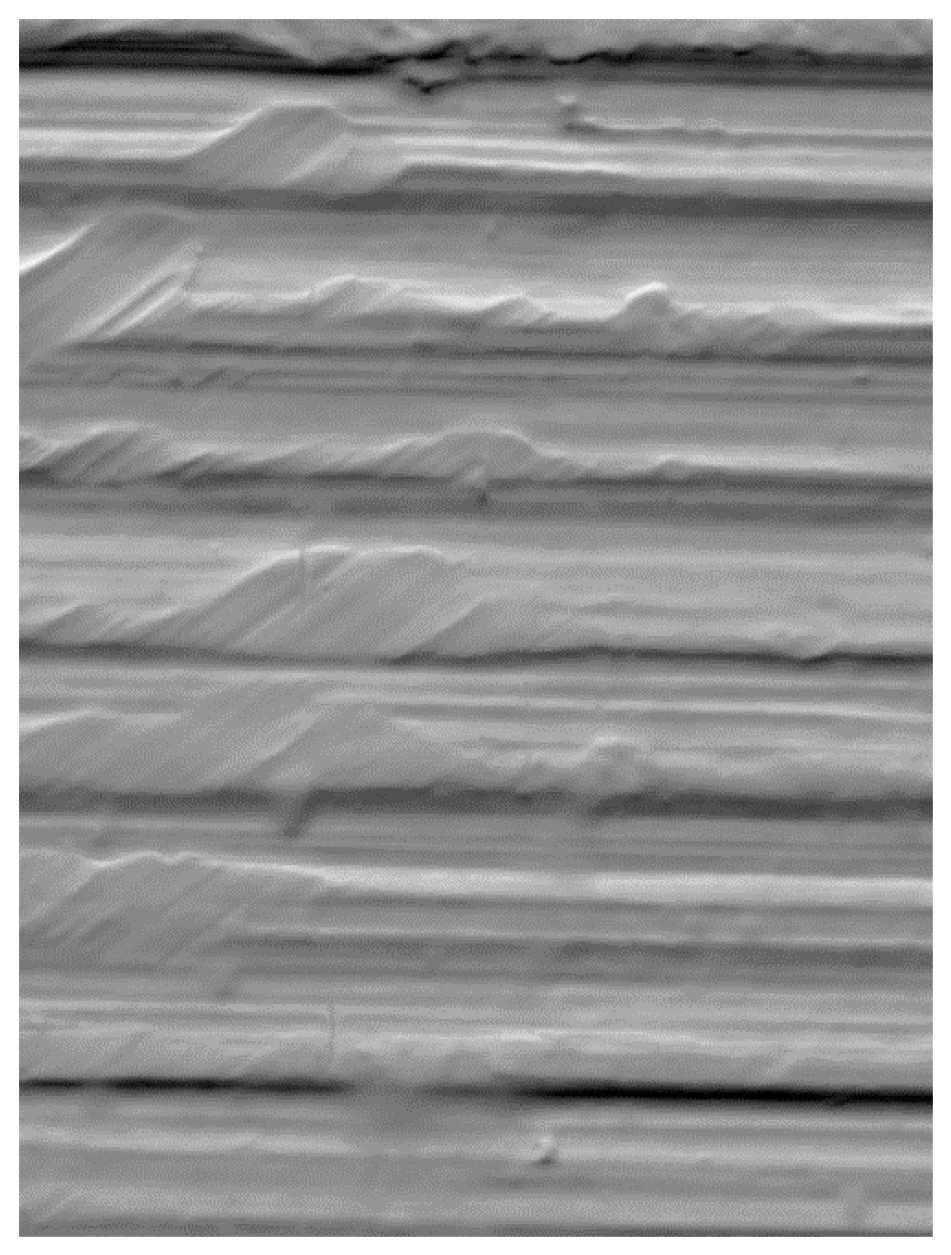
SEM image of the abutment before tightening to the implant.

**Figure 14:**
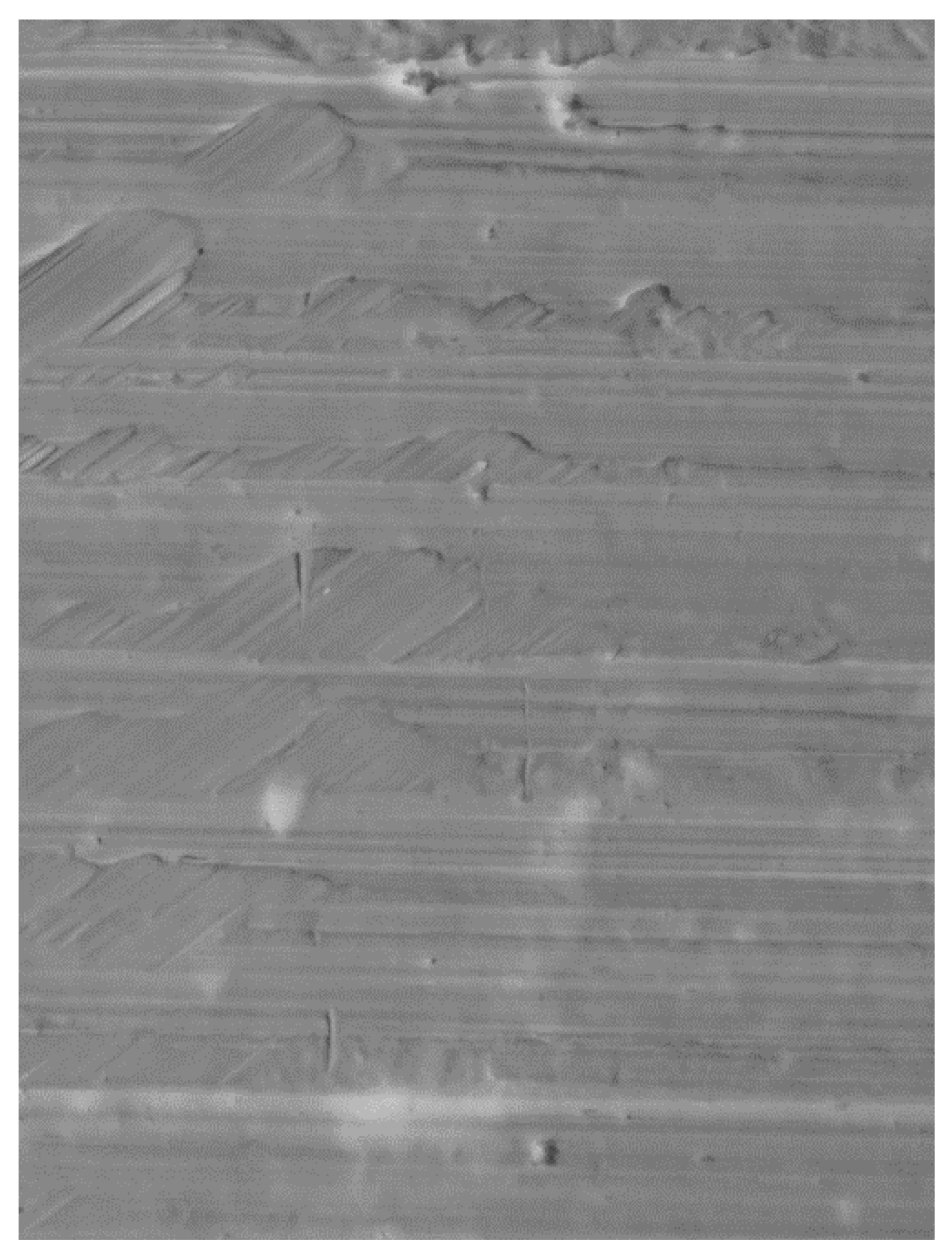
SEM image of the abutment after tightening to the implant.

Figure 13 and Figure 14 show SEM images of the abutments before and after tightening to the implant. The post-tightening image shows discernible smoothing of the surface asperities.

Table 3 present the differences in surface roughness between implant platform and abutment before screwing them together. At baseline, the implant platform was significantly rougher than the abutment (*p* < 0.001). The difference in Ra was 0.34 µm, corresponding to a value nearly 78% higher for the implant platform. The implant-platform Rv was 0.678 µm greater than the abutment Rv. These baseline differences are consistent with the description by Kalpakjian & Schmid (2014) [3] that machining of intraosseous components may preserve microrelief for mechanical engagement, whereas the abutment is finished to a smoother surface.

Rp did not differ significantly between the implant platform and abutment at baseline (*p* = 0.063). Thus, despite differences in overall roughness, the study did not detect a baseline difference in maximum peak height. The implications of this finding for stress distribution were not evaluated.

The profile-shape parameters also differed at baseline. The implant platform showed negative skewness (Rsk = −0.367), consistent with a valley-dominated profile, whereas the abutment showed nearly symmetric skewness (Rsk = 0.053). The lower implant-platform kurtosis (Rku = 1.92) indicates a flatter height distribution than that of the abutment.

After tightening, the implant platform remained significantly rougher than the abutment (*p* < 0.001). The difference in Rv decreased from 0.678 µm at baseline to 0.247 µm after tightening. This numerical convergence indicates a smaller between-component difference in maximum valley depth but does not, by itself, confirm microgap filling or plastic deformation.

After tightening, the Rsk values of the implant platform (−0.142) and abutment (−0.147) were numerically similar. The accompanying text reports no significant difference (*p* = 0.966), but Table 4 lists *p* = 0.002 for Rsk; this discrepancy requires confirmation. If the non-significant comparison is confirmed, the numerical similarity may indicate convergence in skewness, but it would not establish a hermetic interface.

After tightening, the abutment had a higher Rku value (3.790) than the implant platform (2.503). The accompanying text reports a significant difference (*p* = 0.002), whereas Table 4 lists *p* = 0.966 for Rku; this discrepancy requires confirmation. The observed Rku values describe differences in the profile-height distribution but do not, by themselves, demonstrate sharpening or wedging of microtips.

## 4. Discussion

The experimental data indicate that tightening of the tapered implant–abutment connection was associated with significant microgeometric changes at the contact interface. After tightening, the implant-platform Ra was approximately 0.65 µm, which falls within the 0.5–1.0 µm range described by Wennerberg and Albrektsson (2009) [10] for turned or machined surfaces.

The statistically significant reduction in implant-platform Ra (*p* = 0.007), together with the reduction in Rq reported in Table 1 is consistent with the findings of Gehrke et al. (2016) [24] regarding compressive stresses within tapered joints. Their proposed mechanism—localized stress exceeding the elastic limit of titanium micro-asperities and producing permanent plastic deformation at contact zones—may explain the observed roughness changes, but deformation was not directly quantified in the present study.

The change in Rv, which had the largest reported effect size (d = 0.79), may reflect alteration of the implant-platform valley profile after tightening; it does not directly demonstrate interfacial micro-sealing. Quirynen and Bollen (1995) [25] proposed an Ra threshold of 0.2 µm for plaque retention; the post-tightening value in the present study (0.65 µm) remained above this threshold. Because leakage and bacterial colonization were not measured, the biological or sealing implications of this value cannot be determined from the present data.

Ra variability increased after torque application, as shown by the increase in SD from 0.059 µm to 0.17 µm. This finding indicates greater dispersion of the post-tightening measurements and may suggest nonuniform microgeometric adaptation. Manufacturing micro-eccentricity and local substrate-hardness variations are possible explanations, but neither was measured in this study.

Bollen et al. (1996) [26] discussed abutment surface roughness in relation to plaque accumulation and peri-implant tissues. Although the post-tightening implant-platform Ra in the present study was below 0.8 µm, soft-tissue outcomes were not assessed. Similarly, an implant-platform Rku < 3 describes the profile-height distribution but does not establish the absence of surface degradation or delamination wear. The present study did not compare different titanium grades, implant systems, or cost categories.

In a 2022 study [27], morphological changes on the conical implant surface were evaluated after application of a 35 N.cm tightening torque and simulated masticatory loading using scanning electron microscopy (SEM). Implant–abutment engagement was associated with surface modification and plastic deformation of micro-asperities. Metallic (titanium) connections showed controlled and homogeneous micro-adaptation, whereas one-piece zirconia abutments produced more pronounced structural damage to the implant cone, attributed to differences in material hardness. These findings are broadly consistent with the surface-roughness and morphology changes observed in the present study, although loading conditions and materials differed.

Zhai et al. (2022) [28] investigated conical implant–abutment connections with a 6° Morse taper under tightening torques of 25, 30, 35, and 40 N.cm after dynamic fatigue loading. The resin-embedded specimens were sectioned longitudinally and evaluated by scanning electron microscopy (SEM). Increasing torque, particularly between 30 and 35 N.cm, increased the linear contact area and reduced the microgap to below 1 μm. These findings are consistent with the present observations of profile smoothing and convergence in Rv values, but the current study did not directly measure microgaps, sealing, or the effects of different torque levels.

de Morais et al. (2023) [29] examined the effects of abutment-tightening forces on implant-platform deformation using two-dimensional and three-dimensional micro-computed tomography (micro-CT) in narrow- and standard-platform implants. They reported measurable changes in volume and geometry within the implant–abutment connection and found that deformation varied with implant design and manufacturing characteristics. These findings provide a possible context for the increased SD of Ra observed after tightening in the present study. However, manufacturing-related micro-eccentricity and localized geometric variability were not measured here; therefore, they remain hypotheses rather than demonstrated causes.

The current literature provides only limited information regarding the surface roughness of the implant platform and the internal implant connection. Most studies involving scanning electron microscopy focus on microgap assessment, manufacturing defects, surface roughness of the external part of the implant, connection wear, or deformation after mechanical loading. Quantitative evaluation of roughness parameters such as Ra, Rq, Rsk, and Rku on the implant platform and corresponding abutment contact surfaces before and after tightening has rarely been reported. Consequently, the influence of tightening-induced surface modifications on implant–abutment adaptation and sealing remains insufficiently understood.

## 5. Study Limitations

This investigation was conducted under laboratory conditions using a single implant system and a single tightening cycle. Dynamic fatigue loading, repeated assembly/disassembly procedures, and microbiological analyses were not performed. Therefore, the findings should be interpreted within the limitations of an in vitro experimental design.

## 6. Conclusions

Within the limitations of this in vitro study, a single tightening and removal cycle was associated with significant changes in the measured surface-roughness parameters of both the implant platform and abutment. Ra and Rq decreased after tightening for both components, while the patterns of change in Rp, Rv, Rsk, and Rku differed between the implant platform and abutment. Together with the SEM observations, these results indicate microtopographic adaptation of the investigated conical interface under the tested conditions. They do not directly establish the proposed mechanisms of asperity deformation, microgap filling, or interfacial sealing.

Accordingly, the findings support surface micro-restructuring after tightening in the investigated implant system, but they do not demonstrate long-term biomechanical reliability, resistance to bacterial micro-leakage, or clinical benefit. Confirmation of such outcomes would require mechanical-fatigue, leakage, microbiological, and clinical evaluations beyond the scope of this study.

## Figures and Tables

**Table 1:** Statistical comparison of the parameters reflecting the surface roughness of the implant platform before and after tightening to the abutment.

Parameter	Pre- Screwing (Mean ± SD)	Post- Screwing (Mean ± SD)	Change	*p*-Value
Ra (µm)	0.777 ± 0.059	0.650 ± 0.170	decreases	0.007
Rq (µm)	0.895 ± 0.066	0.765 ± 0.185	decreases	0.005
Rv (µm)	1.845 ± 0.157	1.544 ± 0.339	decreases	0.002
Rp (µm)	1.347 ± 0.066	1.444 ± 0.332	no significant change	0.18
Rku	1.920 ± 0.091	2.503 ± 1.577	no significant change	0.118
Rsk	−0.367 ± 0.085	−0.142 ± 0.431		0.047

**Table 2:** Statistical comparison of the parameters reflecting the surface roughness of the abutment before and after tightening to the implant.

Parameter	Initial (Mean ± SD)	Loaded (Mean ± SD)	Change	*p*-Value
Ra (µm)	0.437 ± 0.026	0.291 ± 0.027	decreases	<0.001
Rq (µm)	0.526 ± 0.029	0.394 ± 0.044	decreases	<0.001
Rv (µm)	1.167 ± 0.080	1.297 ± 0.161	increases	0.001
Rp (µm)	1.266 ± 0.183	1.002 ± 0.184	decreases	<0.001
Rku	2.467 ± 0.046	3.790 ± 0.298	increases	<0.001
Rsk	0.053 ± 0.281	−0.147 ± 0.277	decreases	0.039

**Table 3:** Difference in roughness between implant platform and abutment before screwing them together.

Parameter	Implant Platform (Mean ± SD)	Abutment (Mean ± SD)	*p*-Value
Ra (µm)	0.777 ± 0.059	0.437 ± 0.026	<0.001
Rq (µm)	0.895 ± 0.066	0.526 ± 0.029	<0.001
Rv (µm)	1.845 ± 0.157	1.167 ± 0.080	<0.001
Rp (µm)	1.347 ± 0.066	1.266 ± 0.183	0.063
Rku	1.920 ± 0.091	2.467 ± 0.046	<0.001
Rsk	−0.367 ± 0.085	0.053 ± 0.281	<0.001

**Table 4:** Difference in roughness between implant platform and abutment after screwing them together.

Parameter	Implant Platform (Mean ± SD)	Abutment (Mean ± SD)	*p*-Value
Ra (µm)	0.650 ± 0.170	0.291 ± 0.027	<0.001
Rq (µm)	0.765 ± 0.185	0.394 ± 0.044	<0.001
Rv (µm)	1.544 ± 0.339	1.297 ± 0.161	0.006
Rp (µm)	1.444 ± 0.332	1.002 ± 0.184	<0.001
Rku	2.503 ± 1.577	3.790 ± 0.298	0.966
Rsk	−0.142 ± 0.431	−0.147 ± 0.277	0.002

## Data Availability

The data presented in this study are available on request from the corresponding author. The data are not publicly available due to ongoing research related to a doctoral dissertation
